# Nationwide survey on attitudes and perceived barriers toward provision of pharmaceutical care among final year undergraduate pharmacy students in the United Arab Emirates

**DOI:** 10.1371/journal.pone.0246934

**Published:** 2021-02-16

**Authors:** Ali M. Tawfiq, Muaed Jamal Alomar, Nageeb Hassan, Subish Palaian

**Affiliations:** 1 College of Pharmacy and Health Sciences, Ajman University, Ajman, United Arab Emirates; 2 Department of Clinical Sciences, College of Pharmacy and Health Sciences, Ajman University, Ajman, United Arab Emirates; BCS Health & Care Scotland, UNITED KINGDOM

## Abstract

Pharmaceutical care (PC) practice is still limited in the United Arab Emirates. It is crucial to understand pharmacy students’ attitudes and their perceived barriers towards PC provision, to evaluate the effectiveness of theoretical and practical curricula in creating positive attitudes toward PC. This study aims to assess attitudes of final year undergraduate pharmacy students in the United Arab Emirates (UAE), and the barriers perceived by them to practice PC. A cross-sectional survey-based study was conducted in February and March, 2020, involving colleges in UAE offering undergraduate pharmacy programs and having students in their final year. Participants filled a questionnaire covering attitudes’ items, based on the Pharmaceutical Care Attitudes Survey (PCAS), and several perceived barriers. A stratified sample of 193 students participated from six universities, 85% were females, 92.2% and 64.8% completed or engaged in community and hospital pharmacy training respectively, at the time of the study. Attitudes’ items receiving the highest agreement were PC will improve patient health (95.3%), all pharmacists should perform PC (93.3%) and PC would benefit pharmacists (92.7%). However, 44.6% agreed PC is not worth the additional workload. Females showed higher attitudes’ total scores, median (IQR): 55 (51–58) and 52 (49–55.5) for females and males respectively, P = 0.032. Having incomplete courses was also associated with lower scores, median (IQR): 55 (51–58) and 52 (48.5–55.5) for “No” and “Yes” respectively, P = 0.048. Poor image of the pharmacist’s role and lack of private counseling area or inappropriate pharmacy layout were the most perceived barriers, with around 78% agreement. In conclusion, final year undergraduate pharmacy students in the UAE have positive attitudes towards pharmaceutical care. The current curricula may be satisfactory in fostering positive attitudes among students. Poor image of the pharmacist’s role and lack of counseling area or inappropriate pharmacy layout were the main barriers identified, among other barriers.

## Introduction

Traditionally, duties of pharmacists were, to some extent, limited to compounding and dispensing of medicines. Since the early 1990s, the role of pharmacists began to expand and evolve to more patient-oriented duties. The concept of pharmaceutical care (PC) has been introduced as one of the emerging services in the pharmacy field [1]. In 2013, the Pharmaceutical Care Network Europe (PCNE) reviewed PC definitions with the objective to redefine PC. The consensus was to define PC as: “the pharmacist’s contribution to care of individuals in order to optimize medicines use and improve health outcomes” [2]. Pharmaceutical care means that pharmacists should assess, monitor and counsel patients, set objectives, document, and perform a variety of other activities [3], and collaborate with other healthcare providers (HCPs), in order to optimize the patient-oriented service, improve outcomes and patients’ quality of life [4], and offer care alongside the product [5]. Offering PC services by pharmacists in different therapeutic areas demonstrated significant improvement in outcomes, mainly by identifying and preventing drug-related problems, as well as those related to the diseases [4]. PC is widely practiced in the parts of the world with more advanced health settings [6]. However, the situation might be different some Arab countries, in which pharmacists’ orientation is still, somehow, focused on the product [7].

The United Arab Emirates (UAE) is a federation of seven Emirates that is located in the Arabian Peninsula in the Middle East. As of 2019, the population of UAE is estimated to be 9,770,529 [8], representing diverse backgrounds, ethnicities and religions from all over the world, with almost 90% of the population being foreigners from more than 200 nationalities [9]. Healthcare facilities in the UAE are divided between the governmental sector and private sector. There are 143 hospitals (45 governmental and 98 private), 7930 pharmacists (1814 in the governmental sector and 6116 in the private) [10]. The number of pharmacies rose from around 2000 in 2010 [11] to more than 3500 in 2016 [12]. Licensing of HCPs in the UAE is granted by 3 authorities according to the geographical area: Ministry of Health and Prevention (MOHAP), Dubai Health Authority (DHA) and Department of Health (DoH) [13] previously known as the Health Authority Abu Dhabi (HAAD) [14]. Pharmacy education in the UAE began with establishing Dubai Pharmacy College in 1992, then other universities and institutes followed [15]. Currently nine universities offer four to five years Bachelor or Doctor of Pharmacy (PharmD) undergraduate pharmacy programs [16]. The PharmD program is an entry to practice degree with a duration of five years including 6 months internship (Advanced Pharmacy Practice Experience) [16, 17]. Postgraduate degrees in the pharmacy field are offered by several universities, as well as Diploma and Higher Diploma programs [16]. Pharmacy curricula in UAE are in a stage of development and transition from focusing on traditional pharmacy and industry, parallel to regional progress in this aspect, to being more patient-care oriented. More PharmD programs are being established similar to a global and regional trend [16]. Besides, there is more focus on provision and enhancement of core competencies required for patient-oriented practice in pharmacy colleges’ curricula [17–23], with more colleges getting accredited from global bodies such as the Accreditation Council for Pharmacy Education (ACPE) [24]. In fact, the UAE Ministry of Education signed a Memorandum of Understanding (MoU) with the ACPE in order to establish cooperation during the accreditation process of pharmacy programs in UAE institutions [25]. Pharmaceutical care is offered as a standalone theory course or as part of other courses by the majority of pharmacy colleges in UAE [17–23]. PC is also practically introduced in the community pharmacy and hospital pharmacy training, which should be completed prior to graduation. So, graduates of pharmacy from UAE universities are exposed to the concept and practices of pharmaceutical care in theory and practical sessions.

While pharmacists are being involved in providing professional health services and interventions internationally with new roles being introduced [7], their practice in UAE is still “traditional” with basically low provision of PC. As a matter of fact, the majority of community pharmacists in the UAE only counsel the patient about dose and frequency of medicine administration, and they may not monitor or educate about adverse events unless they are asked by the patient [26]. It is suggested that patients in the UAE are dissatisfied with community pharmacy services. They require detailed information on the medicines, information about self-management, tips related to lifestyle and a customized service on a personal level [27]. Some studies stated the insufficiency of drug information sources, inability to access patients’ data, lack of space to offer counseling, lack of time, lack of skills, negative image of pharmacists in the public, as well as financial reasons, as obstacles in the way of establishing the concept of PC [4].

Creating a positive attitude among pharmacists towards PC is the cornerstone in implementing and expanding PC services. It is equally important to nourish the same positive spirit in the soon-to-be pharmacists [28]. Several studies have been conducted globally [4, 28–36] as well as in the Middle East in Saudi Arabia [37, 38], Kuwait [39, 40] and Qatar [40, 41] to assess pharmacy students’ attitudes towards PC, and reported that students included in these studies showed positive attitudes toward PC, with the majority agreeing that pharmacists should perform pharmaceutical care, its practice is valuable, and it will improve patient health. Some of them also identified the major barrier against PC practice as a lack of private counseling areas among a variety of other barriers [38–40].

Undergraduate students of pharmacy in the final year will graduate in a few months, and have studied pharmaceutical care as an independent course or under the umbrella of courses in different names. These students are expected to have the basic knowledge and skills to practice pharmaceutical care since they have already finished PC course and they have finished or are in the middle of their training/internship program [17–23]. Promoting a positive attitude towards PC among students who will soon be practicing will help in expanding the practice of PC in the future [42].

Pharmacy students’ attitudes toward pharmaceutical care has not been studied in UAE to the best of our knowledge, and available information in this regard is very limited. It is crucial to understand the perspective of pharmacy students on pharmaceutical care, as this understanding is valuable to assess the effectiveness of theoretical courses [41] and field training [28] in creating positive attitude toward PC. This evaluation may also lead to curricular analysis, changes and improvement. With the practical exposure through internships, students will observe and report obstacles and barriers against PC provision. Identifying these barriers may lead to recommendations to overcome them, as well as curricular development, in order to enhance PC practice establishment [39].

Therefore, this study assessed final year undergraduate pharmacy students’ attitudes, as well as their perceived barriers, toward the provision of pharmaceutical care in the United Arab Emirates.

## Materials and methods

### Design and setting

A cross-sectional survey-based study was conducted in seven pharmacy colleges in the UAE offering undergraduate pharmacy degrees, and having a batch in their final year. Two universities were excluded, as they did not have final year students yet.

#### Study sample

Final year undergraduate pharmacy students were estimated to be 471 through communication over the phone with the included colleges. By using the formula N = N’/1+N’ (0.05)^2^, where N = sample size, N’ = total number of final year pharmacy students, and assuming 0.05 as the marginal error and a confidence level of 95%, the calculated sample size was 216. A demographically stratified proportionate sample among each college was enrolled using a cluster random sampling method. Dividing the sample size (216) by the total population (471) results in a coefficient (0.4586), multiplying it by the number of students in each college calculates the stratified sample for that particular college.

Upon distributing the questionnaires, we collected information about the number of males and females of final year in each university, and the total number was estimated to be 414 females (87.9%) and 57 males (12.1%). According to that ratio, the investigator tried to collect a proportionate sample from each university by distributing the questionnaire to a similarly proportionate ratio of males and females. We were unable to collect data from one of the included colleges due to the closure of educational institutions in UAE, enforced in March 2020 as a result of the COVID-19 outbreak.

#### Inclusion criteria

The study included final year undergraduate pharmacy students in the UAE, whether they are studying Bachelor of Pharmacy or Doctor of Pharmacy programs, who were willing to participate and sign a consent form.

#### Exclusion criteria

Students of pharmacy programs of Higher Diploma and Masters were excluded. Additionally, students who were not present at the time of distribution, and those who had not finished or were not currently engaged in pharmacy internship/ training were excluded, as well as students from other levels of pharmacy.

#### Data collection tool

A questionnaire (S1 File) was used to collect the data, preceded by distributing a consent form (S2 File). Study objectives and PCNE definition of pharmaceutical care were included in the consent form.

Part I of the study tool included questions about sociodemographic characteristics of participants, in addition to other information. Part II included the 13 items of the standard Pharmaceutical Care Attitudes Survey (PCAS) instrument, which was developed to assess pharmacy students’ attitudes toward PC and tested by Chisholm and Martin in the United States in 1996 [43]. This instrument, or modified versions of it, has been used in global [4, 28, 30–34] and regional [37–41] studies. PCAS items are statements intended to measure attitudes toward PC, to which students will state their extent of agreement on a five-point Likert scale. Scoring of the Likert scale was 5 = strongly agree to 1 = strongly disagree. However, the scoring for item 6 and 13 was reversed, as the statements were negatively phrased.

For part III, participants expressed their agreement or disagreement with 25 perceived barriers for PC provision on a Likert scale as well, with same scoring interpretation of positively worded statements of part II. Those barriers were collected and adapted from several similar works [4, 29, 39, 41, 44, 45], and reviewed by the investigators. Item (26) was a sentence asking the students to narrate any other barriers they could think of.

After obtaining the author’s permission to use the PCAS questionnaire, a validation of the structured tool was conducted because the tool had different parts, and the perceived barriers part was collected from different literature and modified. The structured questionnaire was validated by four experts in PC; two Ajman University (AU) faculty, a community pharmacist, and a pharmacist engaged in the pharmaceutical field. Each expert was given a content validation form with three parts. Part I was a general overview of the tool in the form of a table, for which the first item discussed demographic data, and whether all characteristics of the study were included. The experts would tick (yes) or (no) opposite each statement and could add remarks. The second item focused on the questionnaire, and included the following statements: covers adequate content on PC, questions are arranged in a logical order, language is simple and easy to follow, all items necessary to achieve the objectives of the study are included, and any technical terms that can be replaced by simple terms. The table included questionnaire items’ numbers, against which the experts would tick (relevant, needs modification, or not relevant), with a field to add remarks. The last part of the form included a field for the experts to narrate any other comments they had. A fifth expert with experience in linguistics and research proofread the tool to correct grammar or typo mistakes, and comment on the form’s layout and structure of the statements. The investigators then held a discussion about the comments of the experts and the tool was modified based on this discussion.

A pilot study was conducted during November 2019 as part of the ethical approval process, to check the validity and reliability of the tool by administering the questionnaire to five pharmacy students from AU. Reliability was tested and values for Cronbach’s α were 0.816 and 0.904 for part II and III respectively. Item 23 of the barriers was rephrased as it was not quite understandable to the pilot study participants. Data of these students were not included in the final analysis.

### Ethical approval

Ethical approval was obtained from the Research Ethics Committee of Ajman University (Reference Number: H-F-H-2019-Nov-28, Date: 26^th^ of December, 2019). Anonymity of the responders and confidentiality of their responses were maintained.

### Survey distribution

In the period of February to March 2020, and after getting the required approvals from the respective colleges, final year pharmacy students in six out of the seven eligible colleges were randomly approached at the dates and timings suggested as suitable and convenient for the students by the personnel granting the permission, and was mostly in the short breaks following lectures, laboratories or gatherings. The principal investigator (Master of Clinical Pharmacy student conducting this research as part of his master’s thesis) distributed the questionnaire forms after introducing himself, explaining the purpose and objectives of the study, and emphasizing that participation is voluntary. Students who agreed to participate were given the questionnaire to fill only after signing a consent form. Consent forms were collected separately from the questionnaires to maintain anonymity of data, and questionnaires were filled anonymously and separately.

### Data analysis

Data was entered and analyzed using IBM SPSS (Statistical Package for the Social Sciences) for Windows, build 1.0.0.1327. Results of sociodemographic data were reported as frequencies and percentages. Training duration (days) was reported as mean, median and interquartile ranges (IQRs).

Results of attitudes and perceived barriers were reported as percentages of students strongly agreeing, agreeing, neutral, disagreeing or strongly disagreeing to each statement. Besides, median (IQR) scores of Likert scale for each statement were reported. Then, for the purpose of categorizing the 13 items and creating scales for attitudes’ factors during analysis, three groups or “constructs” were hypothesized, and each of the 13 items of attitudes was classified into one of the three constructs to which it fits more, as suggested in the development of PCAS tool [43]. Professional benefit (PB) construct involved items 4, 5, 7–12 of the PCAS questionnaire, Professional duty (PD) involved items 1–3, and items 6 and 13 were grouped into Return on effort (RE) construct.

Distribution of attitudes and barriers data was assessed by Shapiro-Wilk normality test. Nonparametric tests were performed to analyze students’ attitudes and perceived barriers. To identify the significance of differences in scores of attitudes and barriers among different groups, Mann-Whitney U test was used in comparisons involving two groups (gender, marital status, engagement in a pharmacy-related job, attending a pharmacy-related seminar, having incomplete courses, and engagement in a community pharmacy, hospital pharmacy or pharmaceutical industry training), while the Kruskal-Wallis test was used for comparisons involving more than two groups (age groups, reasons for studying pharmacy, and fields of preference after graduation). Then, post-hoc analysis of groups with significant association was performed using Dunn’s multiple comparisons method. Correlation of scores to age groups and training groups was assessed using Spearman’s test. Analysis of the relationship between students’ responses to the individual items of the attitudes and students’ characteristics was done by performing Mann-Whitney U test for gender, having incomplete courses, and engagement in community and hospital pharmacy training; while Kruskal-Wallis test was used for age categories, reasons for studying pharmacy and fields of interest after graduation. Statistical significance throughout the analysis was determined as P-value <0.05.

## Results

The colleges of pharmacies of the six universities included in the study are located in five out of the seven Emirates: Abu Dhabi, Dubai, Sharjah, Ajman and Ras Al Khaimah. The total number of participants was 193 (89.7% of the calculated sample size). Students of one pharmacy college were excluded as mentioned earlier in the ‘study sample’ section.

The data of students’ attitudes and perceived barriers did not follow a normal distribution pattern (Shapiro-Wilk test, P = 0.000), therefore, nonparametric tests were performed.

### Sociodemographic profile of participants

Analysis of sociodemographic characteristics revealed that the vast majority (85%) of participants were females. Participants were from 22 different nationalities, with mean age of 22.08 years (SD, 1.4; min, 20; max 30). Most of participants (92.2%) were engaged in community pharmacy training. More details can be found in Tables 1 and 2.

**Table 1:** Sociodemographic characteristics of participants.

Characteristics	n (%)
**Gender**	
**Female**	164 (85)
**Male**	29 (15)
**Country of Origin**	
**Arab (non-UAE)**	165 (85.5)
**Non-Arab**	23 (11.9)
**UAE**	5 (2.6)
**Age**	
**≤ 21**	74 (38.3)
**22–23**	94 (48.7)
**> 23**	25 (13)
**Marital status**	
**Married**	6 (3.1)
**Unmarried**	187 (96.9)
**Reason for studying pharmacy**	
**Self-will**	134 (69.4)
**Influence of friends or seniors**	16 (8.3)
**Forced by family**	31 (16.1)
**Others**	12 (6.2)
**Are you currently engaged in a pharmacy-related job?**	
**No**	180 (93.3)
**Yes**	13 (6.7)
**Do you have any incomplete courses/requirements that will postpone your graduation?**	
**No**	176 (91.2)
**Yes**	17 (8.8)
**Have you attended any pharmacy related seminar, symposium, workshop other than academic requirements during your pharmacy studies?**	
**No**	34 (17.6)
**Yes**	159 (82.4)
**What is the field of preference after completion of your Pharmacy degree**	
**Hospital pharmacy**	85 (44)
**Community pharmacy**	19 (9.8)
**Pharmaceutical marketing**	34 (17.6)
**Pharmaceutical industry**	19 (9.8)
**Others**	20 (10.4)
**More than one interest**	16 (8.3)

**Table 2:** Students’ engagement in internship/training.

Internship/Training	n (%)	Mean days	Median (IQR) days
**Community pharmacy**	178 (92.2)	31.2	30 (17.7–40)
**Hospital pharmacy**	125 (64.8)	37.2	30 (15–58)
**Pharmaceutical industry**	82 (42.5)	13.27	14 (10–15)

IQR, Interquartile range.

### Students’ attitudes towards pharmaceutical care

More than 90% of students strongly agreed or agreed that PC movement will improve patient health, all pharmacists should perform PC, PC would benefit pharmacists, preventing and solving drug-related problems should be the primary responsibility of all pharmacists, and PC practice is valuable. However, 44.6% strongly agreed or agreed that “providing pharmaceutical care is not worth the additional workload that it places on the pharmacist”, 31.6% disagreed or strongly disagreed, while around 24% of them were neutral on that point. Table 3 includes more details about students’ attitudes towards PC.

**Table 3:** Students’ attitudes towards pharmaceutical care per PCAS item.

Item	Response, n (%)					Median (IQR) scores
	Strongly agree	Agree	Neutral	Disagree	Strongly disagree	
**1. All pharmacists should perform pharmaceutical care**	132 (68.4)	48 (24.9)	9 (4.7)	3 (1.6)	1 (0.5)	5 (4–5)
**2. Primary responsibility of pharmacists in all health care settings should be to prevent and solve medication-related problems**	126 (65.3)	49 (25.4)	11 (5.7)	6 (3.1)	1 (0.5)	5 (4–5)
**3. Pharmacists primary responsibility should be to practice pharmaceutical care**	111 (57.5)	61 (31.6)	17 (8.8)	2 (1)	2 (1)	5 (4–5)
**4. Pharmacy students can perform pharmaceutical care during their clerkship/internship**	68 (35.2)	86 (44.6)	33 (17.1)	4 (2.1)	2 (1)	4 (4–5)
**5. I think the practice of pharmaceutical care is valuable**	115 (59.6)	59 (30.6)	16 (8.3)	3 (1.6)	0 (0)	5 (4–5)
6. Providing pharmaceutical care takes too much time and effort*	5 (2.6)	27 (14)	56 (29)	62 (32.1)	43 (22.3)	2 (2–3)
**7. I would like to perform pharmaceutical care as a pharmacist practitioner**	85 (44)	76 (39.4)	23 (11.9)	5 (2.6)	4 (2.1)	4 (4–5)
**8. Providing pharmaceutical care is professionally rewarding**	89 (46.1)	76 (39.4)	20 (10.4)	7 (3.6)	1 (0.5)	4 (4–5)
**9. I feel that pharmaceutical care is the right direction for the profession to be headed**	98 (50.8)	62 (32.1)	28 (14.5)	3 (1.6)	2 (1)	5 (4–5)
**10. I feel that the pharmaceutical care movement would benefit pharmacists**	112 (58)	67 (34.7)	11 (5.7)	3 (1.6)	0 (0)	5 (4–5)
**11. I feel that the pharmaceutical care movement will improve patient health**	135 (69.9)	49 (25.4)	9 (4.7)	0 (0)	0 (0)	5 (4–5)
**12. I feel that practicing pharmaceutical care will benefit my professional career as a pharmacy practitioner**	120 (62.2)	53 (27.5)	18 (9.3)	2 (1)	0 (0)	5 (4–5)
13. Providing pharmaceutical care is not worth the additional workload that it places on the pharmacist*	26 (13.5)	60 (31.1)	46 (23.8)	29 (15.0)	32 (16.6)	3 (2–4)

* Scoring for item 6 and 13 is reversed, as the statements were negatively worded.

IQR, Interquartile range.

**Fig 1:**
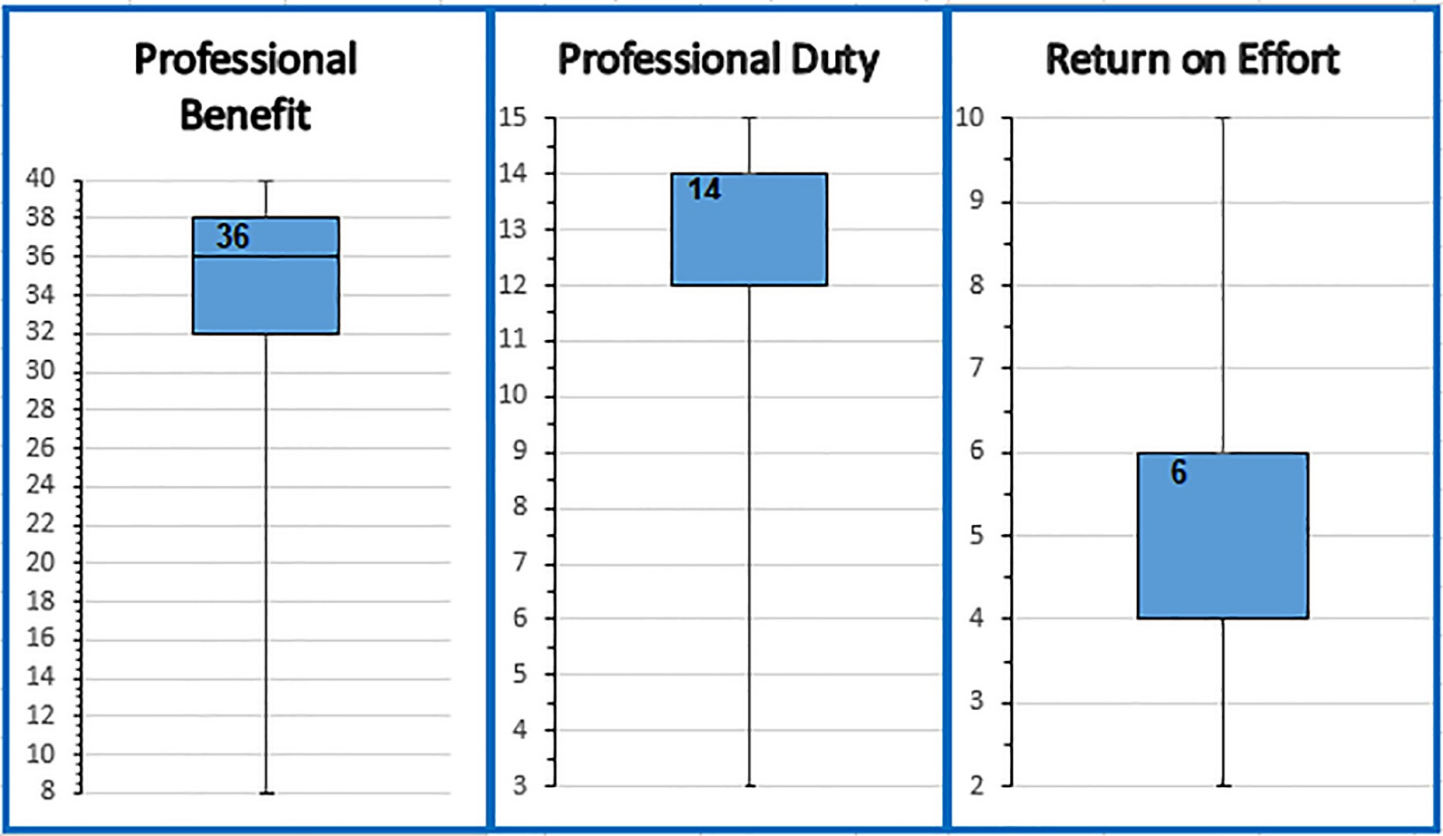
Box plot representation of median (IQR) scores of the constructs of attitudes.

Analysis of students’ attitudes per PCAS scales or “constructs”, explained earlier in the “data analysis” section, showed that the median score for PB was 36 (IQR, 32–38) out of a possible score of 8–40. For PD, the median score was 14 (IQR, 12–14) out of a possible 3–15, while RE median score was 6 (IQR, 4–6) out of 2–10 (Fig 1). Scoring for RE was reversed, as the statements were negatively worded.

#### Further analyses of students’ attitudes

Mann-Whitney U test demonstrated a statistically significant difference in attitudes’ total scores in association with gender (P = 0.032), with median (IQR) total score of 55 (51–58) for females, and 52 (49–55.5) for males. Having incomplete courses/requirements was also associated with significant difference (Mann-Whitney U Test, P = 0.048), for which the median (IQR) for “No” was 55 (51–58), and was 52 (48.5–55.5) for “Yes”. More details can be found in S1 Table.

PB score showed a statistically significant difference associated with gender (Mann-Whitney U test, P = 0.017). Median score for PB was 36 (IQR, 32–38) for females, and 34 (IQR, 31–36) for males. There was a significant association with having incomplete courses/requirements and PD score (Mann-Whitney U test, P = 0.011), for which the median (IQR) scores were 14 (13–15) for the group who did not have incomplete requirements, and 12 (10.5–14.5) for those who had. Engagement in community pharmacy training was also associated with a statistically significant difference in PD (Mann Whitney U test, P = 0.025). Median (IQR) scores were 14 (12–15) for those who were engaged, and 15 (14–15) for the others. (Details in S2 Table). The relationship between individual PCAS items and students’ characteristics was analyzed using Mann-Whitney U test and Kruskal-Wallis test. Female gender, not having incomplete courses or requirements, and not taking community pharmacy training were all associated with significantly higher median scores of several items of attitudes (P<0.05). Details of the items which showed significant association with the characteristics can be found in S3 Table. Assessment by using Spearman’s correlation test showed no statistically significant correlation between PCAS individual items and age groups (P > 0.05). Community and hospital pharmacy training were grouped into 4 groups according to the duration of engagement: “0 days”, “1–20 days”, “21–40 days” and “> 40 days”. Spearman’s test showed weak negative correlation between the statement “I feel that practicing pharmaceutical care will benefit my professional pharmacy career as a pharmacy practitioner” and community pharmacy training duration groups (correlation coefficient, -0.151; P = 0.037).

### Students’ perceived barriers to providing pharmaceutical care

Twenty-three out of twenty-five items were strongly agreed or agreed upon as barriers towards PC by 50% or more of participants (Table 4). More than half of the students (53.4%) strongly agreed that “poor image of the pharmacist’s role in society” is a barrier, with another 25.4% agreeing with that statement. A lack of a private counseling area, space, or inappropriate pharmacy layout was perceived as a barrier by 78.8% of students as well. 38.4% of participants disagreed or strongly disagreed with the statement “Inability to deal with the opposite gender”, 24.4% were neutral, while it received agreement of 37.3%, making it the perceived barrier with the highest disagreement.

**Table 4:** Students’ perceived barriers to pharmaceutical care provision.

Item	Response, n (%)					Median (IQR) scores
	Strongly agree	Agree	Neutral	Disagree	Strongly disagree	
**1. Inadequate drug information resources in the pharmacy**	33 (17.1)	83 (43)	35 (18.1)	35 (18.1)	7 (3.6)	4 (3–4)
**2. Lack of access to the patient medical record in the pharmacy**	50 (25.9)	72 (37.3)	36 (18.7)	27 (14)	8 (4.1)	4 (3–5)
**3. Lack of therapeutic knowledge and clinical problem-solving skills**	39 (20.2)	71 (36.8)	39 (20.2)	35 (18.1)	9 (4.7)	4 (3–4)
**4. Lack of understanding of pharmaceutical care**	42 (21.8)	67 (34.7)	39 (20.2)	37 (19.2)	8 (4.1)	4 (3–4)
**5. Inadequate training in pharmaceutical care**	71 (36.8)	58 (30.1)	34 (17.6)	26 (13.5)	4 (2.1)	4 (3–5)
**6. Lack of private counseling area, space, or inappropriate pharmacy layout**	77 (39.9)	75 (38.9)	18 (9.3)	21 (10.9)	2 (1)	4 (4–5)
**7. Inadequate technology in the pharmacy**	51 (26.4)	62 (32.1)	45 (23.3)	24 (12.4)	11 (5.7)	4 (3–5)
**8. Lack of self-confidence**	37 (19.2)	52 (26.9)	57 (29.5)	30 (15.5)	17 (8.8)	3 (3–4)
**9. Lack of pharmacist time to provide pharmaceutical care**	60 (31.1)	67 (34.7)	37 (19.2)	23 (11.9)	6 (3.1)	4 (3–5)
**10. Poor image of pharmacist’s role in society**	103 (53.4)	49 (25.4)	20 (10.4)	15 (7.8)	6 (3.1)	5 (4–5)
**11. Inadequate (hospital and community pharmacy) internship period**	59 (30.6)	58 (30.1)	40 (20.7)	32 (16.6)	4 (2.1)	4 (3–5)
**12. Negative attitudes of pharmacists towards pharmaceutical care**	47 (24.4)	60 (31.1)	41 (21.2)	40 (20.7)	5 (2.6)	4 (3–4)
**13. Lack of communication skills**	39 (20.2)	68 (35.2)	40 (20.7)	41 (21.2)	5 (2.6)	4 (3–4)
**14. Inadequate staff**	33 (17.1)	69 (35.8)	51 (26.4)	33 (17.1)	7 (3.6)	4 (3–4)
**15. Lack of physicians’ trust in pharmacists’ abilities**	69 (35.8)	67 (34.7)	37 (19.2)	16 (8.3)	4 (2.1)	4 (3–5)
**16. Lack of support from administration**	51 (26.4)	79 (40.9)	49 (25.4)	9 (4.7)	5 (2.6)	4 (3–5)
**17. Absence of legislation for pharmaceutical care**	47 (24.4)	65 (33.7)	56 (29)	20 (10.4)	5 (2.6)	4 (3–4)
**18. Absence of regulations or healthcare policy for pharmaceutical care**	39 (20.2)	66 (34.2)	50 (25.9)	30 (15.5)	8 (4.1)	4 (3–4)
**19. Inability to deal with the opposite gender**	34 (17.6)	38 (19.7)	47 (24.4)	60 (31.1)	14 (7.3)	3 (2–4)
**20. Lack of motive or economic incentive**	44 (22.8)	72 (37.3)	53 (27.5)	20 (10.4)	4 (2.1)	4 (3–4)
**21. Lack of data on the proven value of providing pharmaceutical care**	39 (20.2)	66 (34.2)	58 (30.1)	24 (12.4)	6 (3.1)	4 (3–4)
**22. Pharmaceutical care involves major changes in practice**	55 (28.5)	80 (41.5)	38 (19.7)	20 (10.4)	0 (0)	4 (3–5)
**23. Lack of patient demand and acceptance of pharmaceutical care**	48 (24.9)	66 (34.2)	54 (28)	22 (11.4)	3 (1.6)	4 (3–4)
**24. Resistance from other healthcare providers**	54 (28)	81 (42)	40 (20.7)	17 (8.8)	1 (5)	4 (3–5)
**25. Lack of early exposure of pharmacy students to the principles and practices of pharmaceutical care in pharmacy education**	65 (33.7)	65 (33.7)	34 (17.6)	27 (14)	2 (1)	4 (3–5)

IQR, Interquartile range.

#### Further analyses of students’ perceived barriers

The perceived barriers’ score was significantly associated with engagement in hospital pharmacy (Mann-Whitney U test, P = 0.002); for the engaged group the median score was 94 (IQR 85–104.5), while the group that was not engaged had a median score of 87 (IQR 79–98.75). S4 Table summarizes the perceived barriers vs participants’ characteristics.

Spearman’s correlation test showed a weak negative correlation between community pharmacy training duration groups and some barriers items: absence of legislation, absence of regulations or healthcare policy, lack of therapeutic knowledge and clinical problem-solving skills, inadequate training, lack of self-confidence, and lack of data on the proven value of providing pharmaceutical care. On the other hand, there was a positive correlation between hospital pharmacy training duration groups and absence of legislation, absence of regulations or healthcare policy, lack of access to the patient medical record in the pharmacy, lack of support from administration, and lack of patient demand (S5 Table). A total of 16 students responded to barriers’ item 26 “Any other barriers you can think of”. Some of them mentioned several perceived barriers. Responses were related to economic, financial and motivational barriers (6 responses), insufficient practical training of students (4), communication barriers (3), poor image of pharmacist (2), pharmacists’ negative attitudes (1), insurance issues (1) and lack of a defined job for clinical pharmacists (1).

## Discussion of key findings

The study showed that final year pharmacy students in the UAE have a positive attitude towards the provision of PC. This result is consistent with findings in similar studies in Saudi Arabia [37, 38], Kuwait [39, 40], Qatar [40, 41], the United States [42], Nigeria [30], Poland [35], Nepal [4], Ethiopia [34] and Cuba [28], while Rahim and Nesar [32] reported that pharmacy students in a university in Pakistan had a “moderately positive attitude” toward PC. Our findings suggest that the current curricula of pharmacy colleges in UAE are effective in terms of enhancing positive attitudes of pharmacy students toward PC.

Responses of participants were mostly positive to the items of “professional benefit” and “professional duty” constructs of the PCAS questionnaire, while the two items of “return on effort” received less distinct responses. More than half of the students disagreed that PC takes too much time and effort. Martin and Chisholm [42] also reported disagreement of 67% of respondents in a cross-validation study of the PCAS tool, from analysis of responses of 89 pharmacy students of the second year at the University of Georgia, US. However, other studies [4, 28, 34, 37, 38, 40, 41] observed mostly an agreement with that statement. On the other hand, only one-third of participants in our study disagreed that “providing pharmaceutical care is not worth the additional workload that it places on the pharmacist”, with another quarter giving neutral response, indicating rather negative responses from a considerable number of students. A probable justification may be the absence of incentive or reward schemes that aim to motivate pharmacists to provide more advanced services. Another reason may be that PC is still a new concept in the UAE, taught in universities but not implemented in most of the pharmacy settings yet. The majority of training sites for students offer only the traditional pharmacy services. We expect if students have their internship/ training in settings with well-implemented PC services, they may realize the value of PC, especially the health benefits and impact PC creates on clinical outcomes. The response from students in our study may resemble findings reported by Rahim and Nesar in their study of 200 pharmacy students’ attitudes toward PC in Karachi, Pakistan, as “students answered moderately” to that item. Rahim and Nesar reported that facilities in which students can practice PC are lacking in Pakistan [32]. Similarly, Baral et al in their study of final year undergraduate pharmacy students’ attitudes toward provision of pharmaceutical care in Nepal, reported the agreement of half of the students, and disagreement of around one-third, that PC is not worth the additional workload it places on the pharmacist. Baral et al mentioned a similar situation of lack of focus on PC in pharmacy training sites in Nepal [4]. Around two-thirds of pharmacy students who participated in a study by Ahmed et al [38] in Al-Kharj, Saudi Arabia, also agreed that PC is not worth the additional workload. Tsega et al observed a disagreement of around 40% of participants in their study. Participants included in Tsega’s research have completed 1 year of clinical clerkship with a focus on PC [34]. Other researchers observed higher rates of disagreement to this statement [29, 37, 39–42], indicating more positive responses for that particular point.

Engagement in community or hospital pharmacy training seemed not to influence attitudes’ scores. Nevertheless, we observed, interestingly, that students who were engaged in community pharmacy training responded less positively to the items “All pharmacists should perform pharmaceutical care”, “I think the practice of pharmaceutical care is valuable”, “I feel that the pharmaceutical care movement will improve patient health”, and “providing pharmaceutical care is not worth the additional workload that it places on the pharmacist”. El Hajj et al [41] reported a similar finding, associating more practical experience with lower positive attitude scores. The finding was attributed to the “mismatch” between university curricula and real-life pharmacy practice. Similarly, Udeogaranya et al [30] suggested that pharmacy experience did not improve students’ attitudes toward PC. Ubaka et al [31] conducted a before-and-after study in Nigeria to evaluate the effect of clerkship on students’ attitudes toward PC. They concluded that clinical clerkship courses positively influence PB aspect, but not PD nor RE. On the other hand, Huang et al reported that clerkship will improve students’ understanding and attitudes toward PC, despite required amendments [36]. Our observation was not consistent with these conclusions. In fact, we observed a negative correlation, although weak, between responses to the statement “I feel that practicing pharmaceutical care will benefit my professional pharmacy career as a pharmacy practitioner” and community pharmacy training duration groups.

We observed higher positive attitudes with females compared to males, consistent with findings by Al Arifi (Saudi Arabia) [37] and Udeogaranya et al (Nigeria) [30], while Baral et al [4] did not find a statistically significant difference in attitudes among genders. Upon analyzing gender association with PCAS scales scores, females had significantly higher PB scores, but not PD nor RE.

Although students who were 22–23 years of age had a slightly higher PD score compared to those above 23 years, age did not seem to have a significant influence on students’ attitudes in our study, probably due to the proximity of age among participants.

Students who had incomplete courses or requirements that may delay their graduation had a less positive attitude compared to those who will graduate without delay. It is unclear whether this influence was due to the fact students without incomplete requirements had more courses, theoretical or practical, or due to difference in academic level or grade point average (GPA), implied by having incomplete requirements. Our study was not intended to assess attitudes according to GPA, therefore we did not ask about this information. Baral et al [4] observed an association between having incomplete requirements and the items “Providing PC is professionally rewarding” and “I feel that PC movement will improve patient health”. However, by comparing the results, both groups had similar median scores for both items.

Participants agreed to most of the barriers against provision of PC mentioned in our study. Among these perceived barriers, poor image of pharmacist’s role in society ranked the highest. Other researchers [4, 38, 41] also had similar negative observations regarding pharmacists’ image, while Awaisu et al [40] reported such perception among participants from Kuwait. Pharmacists in the UAE are still trapped in the frame of the traditional role, mostly dispensing. Hasan et al [5] reported that enhanced services were still not offered in majority of pharmacies in the UAE. Offering consultation services has been suggested to improve the image of pharmacists [46]. PC services may help in overcoming the stereotypical image of pharmacists in the society.

A lack of private counseling area, space, or inappropriate pharmacy layout, as well, was highly perceived as a barrier. This is consistent with findings in other countries [4, 38–41]. In a 2017 study [7] conducted in Dubai and Sharjah, only 10% of the surveyed pharmacies had a private counseling area. Having a counseling area is not mentioned among the Licensing of a Pharmaceutical Facility requirements on MOHAP website [47], while DHA Pharmacy Inspection Checklist [48] states the patient consultation area as “optional”. Assigning a private area for counseling purpose may predict quality of counseling [49], help in encouraging patient counseling, and give enough privacy and comfort for the patient.

Resistance from other healthcare providers and lack of physicians’ trust were also highly agreed upon as barriers among our participants. Katoue et al [39] conducted a study on 126 pharmacy students at Faculty of Pharmacy, Kuwait University, and also observed an agreement among more than 70% of respondents that “lack of physicians’ trust in the pharmacists’ abilities”, “lack of communication/coordination with physicians’ and “physicians will not accept pharmacists’ new role” were barriers against PC provision. In 2015, Hasan et al [50] interviewed 53 physicians about their attitudes toward providing primary care services in community pharmacies. Despite showing support to the idea of involving the pharmacist in primary care services, physicians had some reservations about involving the pharmacists in other tasks, stating “concern with pharmacists’ competence, potential ‘conflict of interest’ associated with community pharmacy as a business, patient safety and territorial control” as the rationale for their opinions. Physicians and other HCPs’ attitudes, perceptions and concerns toward provision of PC services by pharmacists are worth further studying. Such studies will help address their concerns or correct any misconceptions. Awareness should be spread among other HCPs about the importance of PC, as well as to identify and correct misconceptions. Including tutorials about PC in curricula of medical, nursing and other relevant colleges may improve other HCPs’ attitude, understanding and perceptions about PC services.

The barrier which received the highest disagreement was “inability to deal with the opposite gender”, with slightly less than 40% disagreeing to the statement, and a similar percentage agreeing. El Hajj and his colleagues [41] observed a higher disagreement to this barrier in their work, of around two-thirds of participants.

Other studies [4, 38–41] reported a lack of access to patients’ medical records, organizational barriers, inadequate training, and inadequate drug information resources as perceived barriers toward provision of PC among pharmacy students. In our study, although to a less extent compared to the previously mentioned barriers, these barriers were also perceived as obstacles in the way of providing PC.

More barriers were perceived by students engaged in hospital pharmacy training, and there was a weak positive correlation between training duration and agreement to the barriers: absence of legislation, absence of regulations or policy, lack of access to medical records, lack of support from administration and lack of patient demand.

Inadequate training in PC, inadequate (hospital and community pharmacy) internship period, and a lack of early exposure to the principles and practices of PC in pharmacy education were perceived as barriers by around two-thirds of students. Theoretical curricula should emphasize the importance of PC early in the journey of pharmacy students. They should also be able to practice it during their training by interacting with, assessing, monitoring and counseling patients [41]. They should be given the chance to identify drug-related problems, document, and interact with other HCPs. Preceptors who supervise the hospital and community pharmacy training should also have deep understanding, knowledge, experience and skills in PC provision, in order to be up to the required standard. Although we did not notice a clear link between attitudes towards PC and practical exposure, well-structured and conducted training courses or internship may enhance the knowledge and skills of pharmacy students required to excel the provision of PC. Assessing knowledge and skills was not in the scope of our study.

### Strengths and limitations

The strength of this study is that it is the first in the UAE, to investigate the attitudes and perceived barriers of pharmacy students toward provision of PC. Besides, it is a nationwide study, including six out of the seven pharmacy colleges having students in final year, and distributed in five of the seven Emirates, thus representing nationwide students. Although exclusion of one college, due to the cancellation of physical classrooms as a result of the Coronavirus (COVID– 19) outbreak, may be a limitation, we believe the participants were highly representative of the final year pharmacy students in UAE. According to our calculations, the sample size was adequate, gender ratio was estimated to be close to the actual ratio, and participants were from diverse origins somehow resembling the actual diversity in UAE universities. Our results may not be generalized to pharmacy students from other levels, diploma or postgraduate pharmacy students. Results may also not be generalized to pharmacy students in other countries. However, there is a global implication in the students’ attitudes and understandings of PC in this study, keeping in mind that participants were from different backgrounds and nationalities which somehow reflects the actual diversity in the UAE. Following graduation, some of these students may work in their countries where pharmaceutical care practice may be different.

The present study had a few limitations. Authors in this research did not compare between attitudes of students in relation to their colleges, nor between Bachelor of Pharmacy (BPharm) and Doctor of Pharmacy (PharmD) programs. The survey tool was a self-report questionnaire filled by students with the presence of the investigator. Therefore, responses may be subject to social desirability bias, suggesting a possibility that students tended to respond in a way that is more desirable rather than their actual opinions, which may be another limitation. The scope of the study was attitudes and perceived barriers towards PC, but did not assess the knowledge and skills required to provide PC.

### Implications for practice

Current curricula of pharmacy colleges in UAE seem effective in nurturing positive attitudes towards PC among students. However, curricula should emphasize the importance of PC early in their journey. Besides, implementation of well-structured training/internship where students can practice pharmaceutical care themselves, under the supervision of competent preceptors, may enhance students’ knowledge and skills required for PC provision and make them perceive the true value and positive impact of pharmaceutical care. At the same time, measures should be taken on the national level to improve the image of pharmacists among other healthcare providers, such as adding tutorials about PC in curricula of other health sciences, as well as spreading awareness about PC among the public. Pharmacies should also accommodate a private space to allow for proper patient counseling.

### Implications for research

For future research, it is important to explore students’ knowledge and skills in relation to PC. Evaluating students’ knowledge, skills and attitudes in relation to PC per different curricula or training programs may give information about the optimal structure of the theoretical and practical PC courses. Exploring practicing pharmacists’ knowledge, attitudes and perceived barriers to PC in UAE can greatly enrich our information about the current situation of pharmacy practitioners, and what plans and measures are required to overcome these barriers and enhance pharmaceutical care practices in UAE.

## Conclusions

Final year undergraduate pharmacy students in the United Arab Emirates showed positive attitudes toward pharmaceutical care. The current theoretical and practical curricula of pharmacy colleges may be satisfactory in terms of fostering positive attitudes among students. Female students and students who did not have incomplete requirements which may delay graduation displayed more positive attitudes. Age did not seem to significantly influence students’ attitudes.

Several barriers against provision of PC were recognized by surveyed students. Poor image of a pharmacist’s role in society and a lack of private counseling area or inappropriate pharmacy layout were the main barriers identified, among other barriers.

## Supporting information

S1 File Questionnaire form. (PDF)

S2 File Consent form. (PDF)

S1 Table Students’ attitudes towards pharmaceutical care vs sociodemographic characteristics. (PDF)

S2 Table Scales of students’ attitudes in relation to sociodemographic variables. (PDF)

S3 Table Relationship between items of students’ attitudes towards pharmaceutical care and sociodemographic characteristics categories. (PDF)

S4 Table Perceived barriers towards pharmaceutical care vs sociodemographic characteristics. (PDF)

S5 Table Correlation between items of students’ perceived barriers towards pharmaceutical care and training categories. (PDF)
