# Supplementary material for: Nationwide survey on attitudes and perceived barriers toward provision of pharmaceutical care among final year undergraduate pharmacy students in the United Arab Emirates
Source: PLoS One. 2021 Feb 16;16(2):e0246934. doi: 10.1371/journal.pone.0246934 (PMC7886123; doi:10.1371/journal.pone.0246934)
Supplement: S1 File — (PDF) [file pone.0246934.s001.pdf]

QUESTIONNAIRE FOR PHARMACEUTICAL CARE ATTITUDE SURVEY (PCAS)

Form No: ☐☐☐☐ **Date:** ☐☐☐☐☐☐☐☐

PART I

SOCIODEMOGRAPHIC CHARACTERISTICS

1.1 University name………….............……College name……………………............………..

Sex: □ Male □ Female

1.2 Country of origin ………………………..

1.3 Age (years) ☐☐

1.4 Reason for studying Pharmacy Degree:

□ Self-will □ Forced by family

□ Influence of friends or seniors □ Others (please specify)…………….

1.5 Marital status

□ Married □Unmarried (single, widowed, divorced)

1.6 Are you currently engaged in any kind of pharmacy related job?

□Yes □No **(go to Q 1.8)**

1.7 What kind of pharmaceutical job are you engaged with?

□ Hospital pharmacy □ Community pharmacy

□ Pharmaceutical marketing □ Pharmaceutical industry

□ Others (please specify)……………..

1.8 Do you have any incomplete courses/ requirements that will postpone your graduation?

□Yes □No

1.9 Have you attended any pharmacy related seminar, symposium, workshop other than academic requirements during your pharmacy studies?

□Yes □No

1.10 What is the field of preference after completion of your Pharmacy degree?

□ Hospital pharmacy □ Community pharmacy

□ Pharmaceutical marketing □ Pharmaceutical industry

□ Others (please specify)……………...

1.11 Where have you done internship during your Pharmacy Degree studies? **(please specify the duration in days in the blank space)**

□ Community pharmacy (……………..days)

□ Hospital pharmacy (……………...days)

□ Pharmaceutical industry (……………..days)

PART II

STUDENT ATTITUDES TOWARDS PHARMACEUTICAL CARE PER PCAS ITEM

Please tick the box that indicates your extent of agreement with the following statements regarding your attitudes towards pharmaceutical care in context of the United Arab Emirates.

No.	Item	Strongly Agree	Agree	Neutral	Disagree	Strongly Disagree
2.1	All pharmacists should perform pharmaceutical care					
2.2	Primary responsibility of pharmacists in all health care settings should be to prevent and solve medication-related problems					
2.3	Pharmacists primary responsibility should be to practice pharmaceutical care					
2.4	Pharmacy students can perform pharmaceutical care during their clerkship/internship					
2.5	I think the practice of pharmaceutical care is valuable					
2.6	Providing pharmaceutical care takes too much time and effort					
2.7	I would like to perform pharmaceutical care as a pharmacist practitioner					

No.	Item	Strongly Agree	Agree	Neutral	Disagree	Strongly Disagree
2.8	Providing pharmaceutical care is professionally rewarding					
2.9	I feel that pharmaceutical care is the right direction for the profession to be headed					
2.10	I feel that the pharmaceutical care movement would benefit pharmacists					
2.11	I feel that the pharmaceutical care movement will improve patient health					
2.12	I feel that practicing pharmaceutical care will benefit my professional career as a pharmacy practitioner					
2.13	Providing pharmaceutical care is not worth the additional workload that it places on the pharmacist					

PART III

STUDENTS PERCEIVED BARRIERS FOR PHARMACEUTICAL CARE PROVISION

Please tick the box that indicates your extent of agreement with the following as barriers for the pharmaceutical care provision in UAE.

No.	Item	Strongly Agree	Agree	Neutral	Disagree	Strongly Disagree
3.1	Inadequate drug information resources in the pharmacy					
3.2	Lack of access to the patient medical record in the pharmacy					
3.3	Lack of therapeutic knowledge and clinical problem-solving skills					
3.4	Lack of understanding of pharmaceutical care					
3.5	Inadequate training in pharmaceutical care					
3.6	Lack of private counseling area, space, or inappropriate pharmacy layout					
3.7	Inadequate technology in the pharmacy					
3.8	Lack of self-confidence					
3.9	Lack of pharmacist time to provide pharmaceutical care					
3.10	Poor image of pharmacist's role in society					
3.11	Inadequate (hospital and community pharmacy) internship period					
3.12	Negative attitudes of pharmacists towards pharmaceutical care					

No.	Item	Strongly Agree	Agree	Neutral	Disagree	Strongly Disagree
3.13	Lack of communication skills					
3.14	Inadequate staff					
3.15	Lack of physicians' trust in pharmacists' abilities					
3.16	Lack of support from administration					
3.17	Absence of legislation for pharmaceutical care					
3.18	Absence of regulations or healthcare policy for pharmaceutical care					
3.19	Inability to deal with the opposite gender					
3.20	Lack of motive or economic incentive					
3.21	Lack of data on the proven value of providing pharmaceutical care					
3.22	Pharmaceutical care involves major changes in practice					
3.23	Lack of patient demand and acceptance of pharmaceutical care					
3.24	Resistance from other healthcare providers					
3.25	Lack of early exposure of pharmacy students to the principles and practices of pharmaceutical care in pharmacy education					
3.26	Any other barriers you can think of:					

Thank you for your participation!
