# Supplementary material for: Nationwide survey on attitudes and perceived barriers toward provision of pharmaceutical care among final year undergraduate pharmacy students in the United Arab Emirates
Source: PLoS One. 2021 Feb 16;16(2):e0246934. doi: 10.1371/journal.pone.0246934 (PMC7886123; doi:10.1371/journal.pone.0246934)
Supplement: S2 File — (PDF) [file pone.0246934.s002.pdf]

Ajman University
Consent Form for Participation in a Scientific Research Project

KINDLY READ THIS FORM VERY CAREFULLY. FEEL FREE TO ASK ANY QUESTIONS YOU MAY HAVE BEFORE AGREEING TO BE PART OF THE STUDY.

You are being asked to take part in a research project aiming at: **assessing the attitudes and perceived barriers of final year undergraduate pharmacy students towards provision of Pharmaceutical Care (PC) services in the United Arab Emirates**.

Pharmaceutical Care is defined by the Pharmaceutical Care Network Europe (PCNE) as: "the pharmacist's contribution to the care of individuals in order to optimize medicines use and improve health outcomes". *

The project is conducted by Ali M. Tawfiq (post-graduate student) working under the supervision of Dr Muaed Alomar (faculty member) working at Ajman University.

Your participation in this research study is voluntary. Your decision whether to or not to participate will not affect your current or future relations with any personnel at AU or relevant to the study. If you decide to participate, you are free to withdraw at any time without any consequences.

- You will be asked to fill a questionnaire.
- All information obtained from you will remain confidential; the research team will use a coding system where your name and personal information will be replaced by numbers so as to ensure full confidentiality. No information about you, or provided by you as a participant in this research project will be disclosed to any other entity (whether public or private) and for whatever reason unless they are directly involved in the named project.
- No risks, physical or otherwise, can be foreseen.
- There is no direct benefit to you for your participation; however, we hope that the information to be obtained from this study will enhance our understanding of the attitudes and perceived barriers of final year undergraduate pharmacy students towards provision of PC services in UAE. This should hopefully contribute to the development of PC services, as well as better curricula.

I have read the above information. I have been given an opportunity to ask questions and my questions have been answered to my satisfaction. I agree to participate in this research.

Name:

Signature: ____________________Date: ___________________________

* Allemann SS, van Mil JF, Botermann L, Berger K, Griese N, Hersberger KE. Pharmaceutical care: the PCNE definition 2013. International journal of clinical pharmacy. 2014 Jun 1;36(3):544-55.
