# Supplementary material for: Nationwide survey on attitudes and perceived barriers toward provision of pharmaceutical care among final year undergraduate pharmacy students in the United Arab Emirates
Source: PLoS One. 2021 Feb 16;16(2):e0246934. doi: 10.1371/journal.pone.0246934 (PMC7886123; doi:10.1371/journal.pone.0246934)
Supplement: S1 Table — (PDF) [file pone.0246934.s003.pdf]

S1 Table. Students' attitudes towards pharmaceutical care vs sociodemographic characteristics

Characteristics	Median attitudes score (IQR)	P-value
**Gender**		
**Female**	55 (51-58)	0.032[a*]
**Male**	52 (49-55.5)	
**Age groups**		
**≤ 21**	55 (50-58)	0.527[b]
**22-23**	55 (51-58)	
**> 23**	54 (50-57)	
**Marital status**		
**Married**	54.5 (50.5-59.25)	0.792[a]
**Unmarried**	55 (50-58)	
**Reason for studying pharmacy**		
**Self-will**	55 (51-58)	0.232[b]
**Influence of friends or seniors**	52 (48.25-57)	
**Forced by family**	54 (48-57)	
**Others**	52 (50.25-58.5)	
**Are you currently engaged in a pharmacy-related job?**		
**No**	55 (50-58)	0.08[a]
**Yes**	51 (51-54.5)	
**Do you have any incomplete courses/requirements that will postpone your graduation?**		
**No**	55 (51-58)	0.048[a*]
**Yes**	52 (48.5-55.5)	
**Have you attended any pharmacy related seminar, symposium, workshop other than academic requirements during your pharmacy studies?**		

**No**	53.5 (48-57.25)	0.12[a]
**Yes**	55 (51-58)	
**What is the field of preference after completion of your Pharmacy degree**		
**Hospital pharmacy**	55 (51.5-58)	0.321[b]
**Community pharmacy**	55 (50-59)	
**Pharmaceutical marketing**	52.5 (49.75-57)	
**Pharmaceutical industry**	53 (47-59)	
**Others**	55.5 (50.25-57)	
**More than one interest**	56 (53.25-60)	
**Engaged in community pharmacy internship/training**		
**No**	57 (49-58)	0.305[a]
**Yes**	54 (50-58)	
**Engaged in hospital pharmacy internship/training**		
**No**	55.5 (50.25-57.75)	0.602[a]
**Yes**	54 (50-58)	
**Engaged in pharmaceutical industry internship/training**		
**No**	55 (51-58)	0.196[a]
**Yes**	54 (49-57.25)	

[a] Mann-Whitney U test

[b] Kruskal-Wallis test

[*] Significant (P<0.05)

IQR, Interquartile range
