# Supplementary material for: Nationwide survey on attitudes and perceived barriers toward provision of pharmaceutical care among final year undergraduate pharmacy students in the United Arab Emirates
Source: PLoS One. 2021 Feb 16;16(2):e0246934. doi: 10.1371/journal.pone.0246934 (PMC7886123; doi:10.1371/journal.pone.0246934)
Supplement: S2 Table — (PDF) [file pone.0246934.s004.pdf]

S2 Table. Scales of students' attitudes in relation to sociodemographic variables

Characteristics	Professional benefit		Professional duty		Return on effort	
	**Median (IQR)**	**P-value**	**Median (IQR)**	**P-value**	**Median (IQR)**	**P-value**
**Gender**						
**Female**	36 (32-38)	0.017[a*]	14 (13-15)	0.069[a]	6 (4-7)	0.386[a]
**Male**	34 (31-36)		13 (12-15)		6 (4-7.5)	
**Age groups**						
**≤ 21**	35.5 (32-38)	0.323[b]	14 (12-15)	0.023[b,c*]	6 (5-7)	0.118[b]
**22-23**	36 (32.75-38.25)		15 (13-15)		5 (4-6.25)	
**> 23**	35 (31.5-37)		14 (12-14.5)		6 (4.5-7)	
**Marital status**						
**Married**	35.5 (32.75-37.25)	0.794[a]	14 (12.25-15)	0.851[a]	5.5 (5-9.25)	0.333[a]
**Unmarried**	36 (32-38)		14 (12-15)		6 (4-7)	
**Reason for studying pharmacy**						
**Self-will**	36 (33-38)	0.117[b]	14 (13-15)	0.075[b]	6 (4-7)	0.445[b]
**Influence of friends or seniors**	33.5 (30.25-36.75)		14 (12-15)		6.5 (4.25-7.75)	
**Forced by family**	35 (31-37)		14 (12-15)		5 (4-7)	
**Others**	36.5 (30.75-39)		13 (11-14)		5.5 (4-7)	
**Are you currently engaged in a pharmacy-related job?**						
**No**	36 (32-38)	0.409[a]	14 (12-15)	0.350[a]	6 (4-7)	0.113[a]
**Yes**	35 (31-36)		14 (12-15)		5 (3-6)	
**Do you have any incomplete courses/requirements that will postpone your graduation?**						
**No**	36 (32-38)	0.108[a]	14 (13-15)	0.011[a*]	6 (4-7)	0.847[a]
**Yes**	34 (30-36)		12 (10.5-14.5)		5 (4-7)	

**Have you attended any pharmacy related seminar, symposium, workshop other than academic requirements during your pharmacy studies?**						
**No**	34.5 (31-37.25)	0.131[a]	14 (12-15)	0.779[a]	5 (4-6)	0.159[a]
**Yes**	36 (32-38)		14 (12-15)		6 (4-7)	
**What is the field of preference after completion of your Pharmacy degree**						
**Hospital pharmacy**	36 (33-38)	0.520[b]	14 (12-15)	0.897[b]	6 (4.5-7)	0.450[b]
**Community pharmacy**	36 (32-38)		14 (12-15)		5 (4-6)	
**Pharmaceutical marketing**	35 (30-37)		14 (12.75-15)		6 (3.75-7)	
**Pharmaceutical industry**	36 (31-38)		14 (12-15)		5 (4-6)	
**Others**	36.5 (30-38.75)		14 (12.25-15)		5.5 (4-6.75)	
**More than one interest**	36.5 (34-38.75)		15 (13-15)		6 (5.25-8)	
**Engaged in community pharmacy internship/training**						
**No**	38 (31-40)	0.092[a]	15 (14-15)	0.025[a*]	4 (2-7)	0.064[a]
**Yes**	35 (32-38)		14 (12-15)		6 (4-7)	
**Engaged in hospital pharmacy internship/training**						
**No**	36 (33-38)	0.584[a]	14 (12-15)	0.520[a]	6 (4-7)	0.702[a]
**Yes**	35 (32-38)		14 (12-15)		6 (4-7)	

[a] Mann-Whitney U test

[b] Kruskal-Wallis test

[c] Post-hoc analysis with Dunn's method demonstrated a significant association between "22-23" group and ">23" group (P=0.046).

[*] Significant (P<0.05)
