# Supplementary material for: Nationwide survey on attitudes and perceived barriers toward provision of pharmaceutical care among final year undergraduate pharmacy students in the United Arab Emirates
Source: PLoS One. 2021 Feb 16;16(2):e0246934. doi: 10.1371/journal.pone.0246934 (PMC7886123; doi:10.1371/journal.pone.0246934)
Supplement: S3 Table — (PDF) [file pone.0246934.s005.pdf]

S3 Table. Relationship between items of students' attitudes towards pharmaceutical care and sociodemographic characteristics categories

Attitude vs characteristic	Category	Median (IQR)	Mean Rank	P-value
**Pharmacists primary responsibility should be to practice pharmaceutical care vs Gender**	F	5 (4-5)	100.11	0.037^{b*}
	M	4 (3-5)	79.4	
**I feel that pharmaceutical care is the right direction for the profession to be headed vs Gender**	F	5 (4-5)	101.75	0.002^{b*}
	M	4 (3-4.5)	70.16	
**I feel that the pharmaceutical care movement would benefit pharmacists vs Gender**	F	5 (4-5)	100.07	0.038^{b*}
	M	4 (4-5)	79.66	
**Pharmacists primary responsibility should be to practice pharmaceutical care vs age category**	22-23	5 (4-5)	106.3	0.038^{a*}
	≤ 21	4 (4-5)	88.18	
	> 23	4 (4-5)	88.14	
**I feel that the pharmaceutical care movement will improve patient health vs age category**	22-23	5 (5-5)	107.77	0.005^{a*}
	> 23	5 (4-5)	86.88	
	≤ 21	5 (4-5)	86.74	
**Pharmacy students can perform pharmaceutical care during their clerkship/internship vs reason for studying pharmacy**	Others	5 (4-5)	128.88	0.003^{a*}
	Forced by family	4 (4-5)	101.55	
	Self-will	4 (4-5)	97.82	
	Influence of friends or seniors	4 (3-4)	57.44	
**Pharmacists primary responsibility should be to practice pharmaceutical care vs Incomplete courses**	No	5 (4-5)	98.74	0.032^{b*}
	Yes	4 (3-5)	78.94	
**I think the practice of pharmaceutical care is valuable vs Incomplete courses**	No	5 (4-5)	99.3	0.035^{b*}
	Yes	4 (3-5)	73.24	
	No	5 (4-5)	99.69	0.018^{b*}

**I feel that pharmaceutical care is the right direction for the profession to be headed vs Incomplete courses**	Yes	4 (3-4.5)	69.18	
**I feel that the pharmaceutical care movement will improve patient health vs Incomplete courses**	No	5 (4-5)	99.23	0.026[b*]
	Yes	4 (4-5)	73.88	
**I would like to perform pharmaceutical care as a pharmacist practitioner vs Field of interest**	More than one interest	5 (4-5)	117.63	0.007[b*]
	Community pharmacy	4 (4-5)	106.03	
	Hospital pharmacy	4 (4-5)	105.84	
	Others	4 (3.25-5)	92.95	
	Pharmaceutical marketing	4 (3-5)	78.49	
	Pharmaceutical industry	4 (3-4)	68.47	
**All pharmacists should perform pharmaceutical care vs Community pharmacy training**	Not taken	5 (5-5)	119.6	0.045[b*]
	Taken	5 (5-5)	95.1	
**I think the practice of pharmaceutical care is valuable vs Community pharmacy training**	Not taken	5 (5-5)	121.9	0.039[b*]
	Taken	5 (4-5)	94.9	
**I feel that the pharmaceutical care movement will improve patient health vs Community pharmacy training**	Not taken	5 (5-5)	119.87	0.039[b*]
	Taken	5 (4-5)	95.07	
**Providing pharmaceutical care is not worth the additional workload that it places on the pharmacist vs Community pharmacy training**	Taken	3 (2-4)	99.69	0.018[b*]
	Not taken	2 (1-4)	65.16	

[a] Mann-Whitney U test

[b] Kruskal-Wallis test

[*] Significant (<0.05)

IQR, Interquartile range
