# Supplementary material for: Nationwide survey on attitudes and perceived barriers toward provision of pharmaceutical care among final year undergraduate pharmacy students in the United Arab Emirates
Source: PLoS One. 2021 Feb 16;16(2):e0246934. doi: 10.1371/journal.pone.0246934 (PMC7886123; doi:10.1371/journal.pone.0246934)
Supplement: S4 Table — (PDF) [file pone.0246934.s006.pdf]

S4 Table. Perceived barriers towards pharmaceutical care vs sociodemographic characteristics

Characteristics	Median barriers score (IQR)	P-value
**Gender**		
**Female**	91.5 (82.25-102.75)	0.364[a]
**Male**	96 (82.5-104)	
**Age groups**		
**≤ 21**	91.5 (81.5-105)	0.350[b]
**22-23**	91.5 (80.75-100)	
**> 23**	95 (88-105)	
**Marital status**		
**Married**	97 (83.25-109.25)	0.490[a]
**Unmarried**	93 (82-103)	
**Reason for studying pharmacy**		
**Self-will**	92 (80-101.25)	0.235[b]
**Influence of friends or seniors**	89 (83-108.25)	
**Forced by family**	92 (82-100)	
**Others**	103 (93.5-105)	
**Are you currently engaged in a pharmacy-related job?**		
**No**	91 (82-102.75)	0.096[a]
**Yes**	97 (92.5-105)	
**Do you have any incomplete courses/requirements that will postpone your graduation?**		
**No**	93 (82-103)	0.472[a]
**Yes**	91 (84.5-95.5)	
**Have you attended any pharmacy related seminar, symposium, workshop other than academic requirements during your pharmacy studies?**		
**No**	92.5 (81.75-103.25)	0.919[a]

**Yes**	93 (83-102)	
**What is the field of preference after completion of your Pharmacy degree**		
**Hospital pharmacy**	90 (80.5-101)	0.569[b]
**Community pharmacy**	94 (79-110)	
**Pharmaceutical marketing**	92 (80-105)	
**Pharmaceutical industry**	91 (85-99)	
**Others**	94.5 (82.75-104.75)	
**More than one interest**	98 (92.25-103.75)	
**Engaged in community pharmacy internship/training**		
**No**	96 (71-105)	0.969[a]
**Yes**	93 (83-102)	
**Engaged in hospital pharmacy internship/training**		
**No**	87 (79-98.75)	0.002[a*]
**Yes**	94 (85-104.5)	

[a] Mann-Whitney U test

[b] Kruskal-Wallis test

[*] Significant (<0.05)

IQR, Interquartile range
