# Supplementary material for: Nationwide survey on attitudes and perceived barriers toward provision of pharmaceutical care among final year undergraduate pharmacy students in the United Arab Emirates
Source: PLoS One. 2021 Feb 16;16(2):e0246934. doi: 10.1371/journal.pone.0246934 (PMC7886123; doi:10.1371/journal.pone.0246934)
Supplement: S5 Table — (PDF) [file pone.0246934.s007.pdf]

S5 Table. Correlation between items of students' perceived barriers towards pharmaceutical care and training categories

Item vs training category (Spearman correlation)	Correlation coefficient (r)	P-value
**Absence of legislation for pharmaceutical care vs Community pharmacy training categories**	-0.204	0.004*
**Absence of legislation for pharmaceutical care vs Hospital pharmacy training categories**	0.234	0.001*
**Absence of regulations or healthcare policy for pharmaceutical care vs Community pharmacy training categories**	-0.236	0.001*
**Absence of regulations or healthcare policy for pharmaceutical care vs Hospital pharmacy training categories**	0.211	0.003*
**Lack of therapeutic knowledge and clinical problem-solving skills vs Community pharmacy training categories**	-0.155	0.031*
**Inadequate training in pharmaceutical care vs Community pharmacy training categories**	-0.228	0.001*
**Lack of self-confidence vs Community pharmacy training categories**	-0.181	0.012*
**Lack of data on the proven value of providing pharmaceutical care vs Community pharmacy training categories**	-0.178	0.013*
**Lack of access to the patient medical record in the pharmacy vs Hospital pharmacy training categories**	0.18	0.012*
**Lack of support from administration vs Hospital pharmacy training categories**	0.184	0.01*
**Lack of patient demand vs Hospital pharmacy training categories**	0.163	0.024*

* Significant (<0.05)
